# The Role of Stiffness in Cell Reprogramming: A Potential Role for Biomaterials in Inducing Tissue Regeneration

**DOI:** 10.3390/cells8091036

**Published:** 2019-09-05

**Authors:** Michele d’Angelo, Elisabetta Benedetti, Maria Grazia Tupone, Mariano Catanesi, Vanessa Castelli, Andrea Antonosante, Annamaria Cimini

**Affiliations:** Department of Life, Health and Environmental Sciences, University of L’Aquila, 67100 L’Aquila, Italy

**Keywords:** mechanotransduction, biomaterials, stiffness

## Abstract

The mechanotransduction is the process by which cells sense mechanical stimuli such as elasticity, viscosity, and nanotopography of extracellular matrix and translate them into biochemical signals. The mechanotransduction regulates several aspects of the cell behavior, including migration, proliferation, and differentiation in a time-dependent manner. Several reports have indicated that cell behavior and fate are not transmitted by a single signal, but rather by an intricate network of many signals operating on different length and timescales that determine cell fate. Since cell biology and biomaterial technology are fundamentals in cell-based regenerative therapies, comprehending the interaction between cells and biomaterials may allow the design of new biomaterials for clinical therapeutic applications in tissue regeneration. In this work, we present the most relevant mechanism by which the biomechanical properties of extracellular matrix (ECM) influence cell reprogramming, with particular attention on the new technologies and materials engineering, in which are taken into account not only the biochemical and biophysical signals patterns but also the factor time.

## 1. Introduction 

The ECM exerts a key role in regulating the stem cell fate decisions both during development and in somatic stem cell niche. Adult stem cells show the ability for self-renewal and to produce different cell lineages and are essential for tissue maintenance and repair. Their presence within the adult tissue is insured by a specific microenvironment named niche that comprises soluble signaling factors, cell-cell, and ECM interactions, but also biomechanical properties of ECM, such as the elasticity, viscosity, and nanotopography [1]. Indeed physical ECM factors, particularly the stiffness of the microenvironment, contribute to cell differentiation [2,3].

Cells interact with ECM through integrin heterodimers, composed of distinct α and β subunits [4]. Integrins are transmembrane receptors that bind their targets in the extracellular space with their extracellular portion, while they bind the cellular cytoskeleton with their cytoplasmatic portion, providing a direct link between cells and their environment [1]. The cell-substrate binding generates forces from the cytoskeleton to these adhesive bonds. The stiffness of the substrate regulates the amplitude of these forces, and consequently, ECM determines the cell response. On a stiff substrate, but not on a soft one, cells may generate a large force at the focal adhesion, exerting powerful effects on the lineage specification and commitment, i.e., elastic environments favor differentiation of mesenchymal stem cells (MSC) into adipocytes, while on stiffer substrates osteogenesis is promoted [2]. As best reviewed by Isomursu et al., 2019 the forces from the cytoskeleton to this adhesive bond is influenced by ECM composition, as well as by the expression of particular subsets of integrin heterodimers [1]. Thus, stem cells can perceive the stiffness of ECM, and contextually they reorganize their ECM, creating a local niche. Moreover, they can remodel the ECM adding mechanical heterogeneity. The understanding of the crosstalk between stem cell and ECM could help in designing stem cell-based regenerative approaches and innovative biological substrate for tissue engineering. In this review, we focus our attention on the impact of ECM bio-mechanical properties, such as stiffness, on stem cell behavior, cell reprogramming and on the new strategy for tissue engineering and stem cell-based regenerative therapies. 

**Figure 1:**
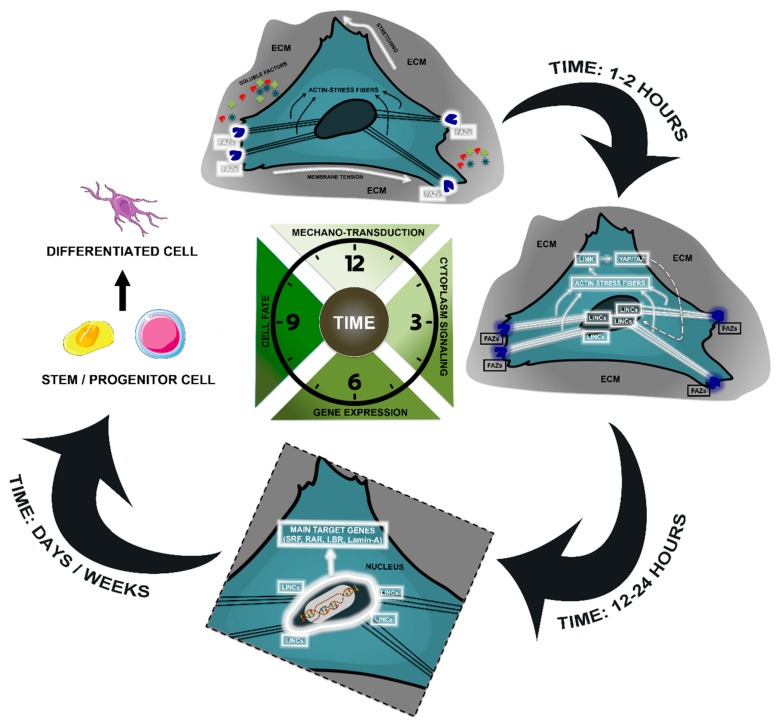
Mechanotransduction converts mechanical stimuli into biochemical signals to modulate cell behavior and function. Generally, the pathways involve receptors at the focal adhesions, mechanosensors, nuclear signaling factors, and nuclear deformation mediated by LINCs and Laminin A, leading to the modulation of gene expression. These phases’ timescale ranges from seconds for the stretching of mechanosensors, hours for alteration in gene expression, days for modification in cell behavior and function, while severe and permanent changes in phenotype, such as differentiation, require weeks.

Tissues present different stiffnesses (defined as Young’s modulus, or elasticity, of a material), i.e., brain tissue is soft (~2500 Pa), while bone tissue is very stiff (~18,000 Pa) (Figure 1) [5,6,7,8]. “Rigid” calcified bone has a very high Young’s modulus and needs very high stress to extend it whereas brain tissue requires very little stress. Moreover, the ECM stiffness in different pathologies results modified, as in scar tissue and tumor samples where it generally has higher stiffness compared to healthy tissue counterparts [5].

Tissue stiffness correlates with the increase of collagen expression, while the hydration state of tissues is inversely proportional to the tissue microelasticity [9]. Tissues subjected to strong mechanical stress, like muscle and bone, have more collagen and are stiff, while tissues that are protected from mechanical stress, such as brain and marrow have low collagen and are soft [9]. In the ECM other matrix components such as proteoglycans and adhesive proteins, through own osmolarity property or interactions with collagens and cells, modulate the mechanical properties of ECM. Matrix stiffness can regulate intracellular signaling pathways important for spreading, intrinsic cellular contractility, cell migration (durotaxis), cell proliferation [10]. 

The property of cells to migrate from softer to stiffer matrix is known as durotaxis [11] for instance durotaxis might direct cancer cells migration [12], as well as the cell migration during embryogenesis [13]. 

Stiffness can regulate cell growth, controlling the apoptosis [14]; i.e., in NIH 3T3 cell line cultivating on soft materials an increase of apoptosis and decrease of proliferation were observed while the opposite was observed on stiff substrates. On a stiff substrate, but not on a soft one, cells may generate a large force at the focal adhesion, exerting powerful effects on the lineage specification and commitment, i.e., elastic environments favor differentiation of MSC into adipocytes, while on stiffer substrates osteogenesis is promoted [2].

## 2. The Mechanisms of Stiffness Sensing

Cells are subject both to external forces (extrinsic forces) and exert forces (intrinsic forces) that are able to activate precise intracellular signaling pathways. Extrinsic forces are applied by shear, tension or compression; while cell-generated forces are intracellular forces that are transferred to the other cells by cell-cell junctions, or through ECM adhesion. These forces are transmitted, through the cell-cell junctions, to the cytoskeleton of another cell. 

Regarding the stiffness the intrinsic forces are proportional to matrix rigidity [15]. In fact, the stiffness of ECM influences the magnitude of these forces and consequently the cells intracellular pathways. 

The mechanism by which cells perceive mechanical forces and translate them into intracellular signals is known as mechanotransduction [16]. Typically, this mechanism involves focal adhesions, mechanosensors and nuclear signaling factors that induce changes in genes and proteins expression profile. These phases’ timescale ranges from seconds for the stretching of mechanosensors, hours for alteration in gene expression, days for modification in cell behavior and function, while severe and permanent changes in phenotype, such as differentiation, require weeks [1,11,12] (Figure 1). 

Cell-ECM interactions are triggered by the integrins, which represent the al link between cells and their environment. Integrins bind their targets in the extracellular space and associate with the cellular cytoskeleton via their cytoplasmic domains, forming focal adhesion zones (FAZs). These adhesion sites interconnect cells with the ECM, and they are composed of multiprotein complexes (adhesome) that allow mechanical coupling [17,18,19,20,21]. Assembly and turnover of this adhesion are affected by the elasticity of the substrate. In fact, stiff substrates stabilize adhesion, while on soft substrates less tensile forces are produced leading to an adhesion destabilization [22,23,24]. The adhesome, through its plasticity, allows the activation of a wide variety of signaling networks [25] and consequently a precise cell response to the internal force generated. As best reviewed by Zaidel-Bar1 and Geiger 2010, the understanding of the adhesome dynamics and plasticity is fundamental to explain the sensitiveness and responsiveness of FAZs to external signaling cues and forces.

The cells test the rigidity of the newly bound matrix by contracting them inducing the activation of myosin II motors [15]. These intrinsic forces produce conformational changes in some adhesion components, such as talin, vinculin, and focal adhesion kinase FAK, and consequently they cause the recruitment and activation of further proteins belonging to mechano-signaling pathways [26]. In particular, the resistance of ECM to cell forces modulates the stability of focal adhesions activating the focal adhesion kinase (FAK), which in turn phosphorylates and activates specific signals, such as Rho kinase (RhoA). The signal pathway activated by RhoA involves the activation of ROCK, which phosphorylates myosin light chain (MLP) generating intrinsic forces [27]. Thus, FAZs sense the mechanical properties of ECM and traduces into the intracellular actin cytoskeleton remodeling producing forces able to, activate multiple mechanosensitive signaling pathways inducing various mechanosensitive transcription factors such as transcriptional coregulators yes-associated protein 1 (YAP), transcriptional coactivator with PDZ binding motif (TAZ), and myocardin-related transcription factors (MRTFs) [28,29]; or to communicate directly the forces from focal adhesion to the nuclear membrane modulating nuclear events, such as transcription.

## 3. Mechanosensitive Signaling Pathways

YAP (Yes-associated protein) and TAZ (Tafazin-associated) are transducers of cell structural characteristics, such as polarity, shape, and cytoskeletal organization that are strictly related to the surrounding ECM. YAP and TAZ are typically in the cytosol of cells growing on soft matrices, but, they translocate into the nucleus when cell contractility enhances on a stiff matrix, apparently independently from Hippo/LATS (large tumor suppressors) signaling pathway [30]. 

These regulations require cellular mechano-transduction, RHO-GTPase signaling, but also inflammation, metabolism, and, instead, contact inhibition and G protein-coupled receptor (GPCR) signaling. Among the RHO family of GTPases, RAS homolog family member A (RHOA), RAS-related C3 botulinum toxin substrate 1 (RAC1), and cell division cycle 42 (CDC42) are the most well-known and they are recognized as pivotal regulators of cytoskeletal dynamics and morphology [31,32]. Adhesion of cells to ECM induces YAP nuclear translocation and activation through F-actin accumulation mediated by RHOA [33]. Notably, RHO/RAC activation induces the formation of F-actin stress fibers by RHO-associated protein kinase (ROCK) and p21-activated kinase (PAK) stimulation that in turn causes LIM kinase-1 (LIMK) activity and inactivate cofilin, leading to F-actin accumulation. F-actin by sequestering angiomotin (AMOT), an inhibitor of YAP, promotes YAP activation and nuclear translocation [34]. Activation of YAP/TAZ is needed for survival of human embryonic stem cells (hESC) [35], growth of uveal melanoma cells [36] and, mesenchymal and neural stem cells differentiation [37,38]. 

Regarding MSC, a reduction in cytoskeleton tension and cell spreading promote the nuclear exclusion of YAP through its phosphorylation determining the adipogenic differentiation [38]. In neural stem cell (NSC) differentiation it has been observed that neurogenic commitment is impaired by a stiff ECM [37]. The Authors demonstrated through a mechanically tunable ECM system that matrix stiffness determines NSC lineage commitment via a YAP and β-catenin interaction during a defined temporal window [37]. Contrary to the proposed model of YAP in regulation in MSCs, in NSC the stiffness did not lead to nuclear translocation of YAP. In NSC stiff matrix promotes the bind of YAP to β-catenin reducing the ability of β-catenin to translocate into the nucleus, and consequently to interact with its target genes, such as proneuronal transcription factor NeuroD1. 

Concerning MRTFs, it is known that their N-terminal RPEL motif can bind G-actin, this binding prevents MRTF’s nuclear localization. The induction of actin polymerization leads G-actin to dissociate from MRTF, inducing the nuclear localization of MRTF [39]. At nuclear level, MRTF binds serum response factor (SRF), and the heterodimer binds the CC(A/T)-richGG cis-element in the promoter region of their target genes [40,41,42], such as genes encoding for cytoskeletal proteins. MRTF translocation induced by mechano-transduction has pivotal roles in a phenotypic switch such as epithelial–mesenchymal/–myofibroblast (EMT/EMyT) [43,44,45] and fibroblast–myofibroblast transition [46,47,48].

Finally, actin network can communicate the forces from FAZ in the plasma membrane to the nuclear membrane at the level of Linker of Nucleo-skeleton and Cytoskeleton (LINC) complexes that transduce mechanical force into the nucleus, modulating nuclear events, such as transcription (Figure 1).The LINC complex is composed of nesprins spanning the external nuclear membrane and binding to SUN proteins, that reach the internal nuclear membrane. Finally, SUN proteins, in turn, engage the underlying Lamina proteins [49,50] that directly regulate transcription factors such as serum response factor (SRF) and retinoic acid receptors (RAR) and indirectly regulate chromatin remodeling through the interaction with the Lamin-B receptor (LBR) [51]. Lamins dictate the mechanical features of the nucleus [52] and LaminA/B ratio is modulated by ECM stiffness [9]. Lamin A/B ratio increases systematically with tissue stiffness, with high levels of Lamin-A and Lamin-B in stiff and in soft tissues, respectively. Mainly, Lamin-A is induced by mechanical stress during differentiation, i.e., in MSCs osteogenic differentiating conditions there is a strong increase of endogenous Lamin-A, while the adipogenic conditions slightly suppressed Lamin-A levels; moreover, a stiff matrix in concert to Lamin-A overexpression led to an increase of positive cells for a standard marker of osteogenesis [9]. Lamin-A knockout results in SRF suppression, which is able to induce adipogenic differentiation [52]. In MSCs adipogenic stimuli induced by a soft matrix, with partial knockdown of Lamin-A cause an increase of in vitro adipogenesis. Moreover, a soft matrix favors Lamin-A phosphorylation and turnover, and low Lamin-A levels decreased its transcription by modulating the nuclear localization of RA transcription factors increasing cytoplasmic levels of RAR [9].

Differentiated cells display a close relation between cell cytoskeleton and genomic programs [53]. This is facilitated by an intricate network of physical links between the cell and nuclear membranes consisting of cytoskeletal proteins [54]. The nuclear membrane is bound to chromatin by Lamins thus anchoring chromatin [55]. The spatial organization of chromatin is further defined by a series of transcription factors and chromatin remodeling enzymes [56,57]. Recent works have shown that changes in cellular architecture have crucial roles in gene expression. 

In cell microenvironment, this modulation may occur by local mechanical stimuli and specific signaling pathways by growth factors and cytokines. It has been shown that signals received at the plasma membrane were transduced to the cell nucleus via both physical and biochemical signals tuning gene expression [58]. As a consequence, cells may change their behavior and even start trans-differentiation pathways, such as the mesenchymal-to-epithelial transition [59]. Such changes in cell fate are devoted to maintaining tissue homeostasis, but when deregulated, they may lead to disease initiation [60].

The cellular cytoskeleton is also crucial for the maintenance of the packing of the chromatin in the nucleus that controls cell type-specific gene expression.

In agreement with the link between chromatin conformation and gene expression, recent works, studying modifications in the cellular cytoskeleton and nuclear architecture using micropatterned substrates, have demonstrated modifications in gene expression in response to the imposed geometry of the substrate dictating cell architecture alterations [61]. For instance, Wang and co-Authors demonstrated that culturing fibroblasts on high polarized geometries induced the expression of matrix genes, while the same cells on more relaxed geometries expressed cell cycle-related proteins [62].

## 4. Biomaterials

In tissue engineering, the features for biomaterial are well-defined. Biocompatibility is pivotal since bioreactor materials, and scaffolds need to be tissue-friendly, without activating immunoresponse [63]. Furthermore, the biomaterials should support tissue and cellular functions, including differentiation, proliferation, and adhesion thanks to its characteristic surface chemistry [64]. Controlled biodegradation is a critical issue that needs to be considered as well [65]. 

Biomaterials defined as “a non-viable material used in a medical device, intended to interact with biological systems” by European Society of Biomaterials [66] can be divided into natural and synthetic materials. 

Natural biomaterials can be classified in protein-based biomaterials such as collagen-, fibrin, elastin, silk-based material scaffolds and polysaccharide materials such as hyaluronic acid, agarose, chitosan, and alginate [67]. 

The advantages of naturally derived biomaterials are that they mainly derived from an in vivo source; consequently huge quantities are regularly available at a reasonable price [68]. Moreover, they show a construction close to the native tissue, and they can be used as reparative or regenerative materials [63,64]. A further advantage is high biocompatibility, due to the existing binding sites for cells and adhesion molecules. However, one of the disadvantages is the natural variability in the in vivo source, and the possible presence of impurities, which can trigger immune reactions [69]. Other problems of natural biomaterials comprise inconsistency in compositions and properties and weak mechanical strength [70]. To overcome these limitations, recent developments in tissue engineering fabrication have designed biomimetic scaffolds able to incorporate ligands imitating the native ECM. Indeed, these scaffolds are used as analogs of the natural ECM in vitro to help in understanding the intricate interplay between cell and ECM [64,71,72].

Collagen can be generated from many sources: amniotic membrane (AM) represents the most attractive. The ECM components of the AM basement membrane are naive scaffolds for cell seeding in tissue engineering, in particular related to skin, eye, nerve, cartilage [73]. The immune system well tolerates collagen. Collagen is well-preferred as scaffold since it can support a variety of cellular differentiation types [74]. 

Another easily accessible scaffold is fibrinogen, obtained from plasma [75]. Basing on the usage purposes, different methods were developed to create fibrin gels, including the separation of components from the serum of patients or commercially. Fibrin gel is used primarily in tissue engineering as hemostatic plug, wound healing, and scaffold for cell migration and/or proliferation [76]. 

Silks are protein polymers that are spun into fibers by *Lepidoptera larvae*, including silkworms, scorpions, spiders, flies, and mites [77]. Silk protein is a suitable material for nanotechnology, thanks to the high biocompatibility and biodegradability, mechanical stability, self-restructuring and easy control [78]. The silk biopolymer finds valuable uses in tissue regeneration as a matrix of wound healing and as a treatment for burn victims; these peptides are also used in cosmetics [78]. Interestingly, it has been demonstrated that silk helps more intensive chondrogenesis than collagen used as a scaffold material for cartilage engineering [79].

Cellulose is a polysaccharide material extracted from wood and plants. Natural cellulose spheres are frequently applied in immobilized reaction, bioseparation, and cell suspension culture [80].

Chitosan is a partly deacetylated derivative of chitin. Chitin is easily obtained from shrimp shells or crab and fungal [81]. Chitosan is used in place of synthetic polymers in ophthalmological purposes. Indeed, chitosan shows all the properties of an ideal contact lens; including gas permeability, optical clarity, optical correction, biocompatibility and mechanical stability [81]. Chitosan is also used for the repair of articular cartilage [82].

Alginate is a hydrophilic polysaccharide extracted from soil bacteria or marine brown algae (i.e., *Laminaria hyperborea*). The hydrolysis of collagen and alginate-a polysaccharide established with crosslinking agent creates sponges. The sponges were mixed with natural compounds used for burn treatment such as tamanu oil, and it has been shown a better wound healing and prevention to infections in animal models [83,84]. In a clinical trial, calcium-alginate was used to encapsulate pancreatic islet cells. These cells preserved the capability to produce insulin, basing to the requirements of the host as the encapsulation avoid unwanted immune responses against the grafted cells [85].

Synthetic materials have high reproducibility, verifiable degradation, mechanical properties, and good material composition control, all features that may not be present in natural materials [86,87]. Since synthetic materials often do not present cell adhesion sites, it is necessary to create chemical cues for cell growth and adhesion [86]. While natural biomaterials have a similar native tissue composition, they better allow cell growth and adhesion [67]. The combination of both natural and synthetic polymers, mostly into the 3D structures, prevents the use synthetic or natural materials alone [67].

Among synthetic materials, we may list: Polymers, ceramics, metals, and graphene. Synthetic polymers approved by the US Food and Drug Administration (FDA) for clinical applications are polyglycolic acid (PGA), poly-L-lactic acid (PLLA), copolymer polylactic-co-glycolic acid (PLGA), poly (ɛ-caprolactone) (PCL) and poly-ethylene glycol (PEG) [86]. Ceramic-based biomaterials include bioactive glasses, calcium phosphates, and hydroxyapatite; they are inorganic non-metallic solid made up of metal or non-metal compounds, which are assembled at high temperatures [86]. Hydroxyapatite, the inorganic compound of bone, is used as a porous scaffold. It is combined with other biocompatible, organic polymers, such as collagen, PLGA or chitosan [88]. This combined biomaterials also improve the drug delivery capacity of these scaffolds to induce bone formation [88,89,90].

Among metal-based biomaterials, chromium, titanium, and tantalum are to mention. The high durability of metals is exploited especially in joint prostheses, hearth valves and dental implants [91,92]. These metals are bioinert but in some cases they induce immunological responses, including allergies. Another disadvantage is that they are not biodegradable [93,94]. 

Recently, among synthetic materials, graphene and its derivatives, including reduced graphene oxide (rGO), graphene oxide (GO), and graphene-based nanostructures have proven to be valid as biomedical approaches [95,96]. Interestingly, it has been reported that rGO can determine the modulation of pathway involved in mechano-transduction and differentiation, by affecting YAP/TAZ localization outside the nuclei and increasing neuronal differentiation markers. This suggests that the mechano-transduction pathways are responsible for the differentiation process [97].

Regarding the application in tissue engineering, the purpose is to detect the natural or synthetic biomaterial or a combination, with the most suitable performance in vivo, able to induce cell differentiation and proliferation and to reestablish the normal architecture of ECM [64].

Combining natural and synthetic materials represents an important strategy in bioengineering [68,98]. These materials are able to mimic the structure, functionality, and morphologies of human tissues as observed in orthopedics and dentistry experimental studies [99]. Unfortunately, there is the paucity of biomaterials and biostructures with clinical applicability.

Decellularization removes cells from tissue, maintaining the biomechanical properties of the ECM, rendering decellularized extracellular matrix (dECM) a suitable biomaterial for tissue regeneration. 3D bioprinting technology is a reproducible and precise technique that allows printing ECM and autologous cells together fabricating patient-derived cell structures mimicking the intrinsic features of natural ECM (extensively reviewed in [100]).

Several agents are utilized for decellularization able to modify ECM composition and structure. The choice of a suitable agent for decellularization is dictated by cellular and lipid content, density, and thickness of the tissue with a slight variation of ECM. Among the different agents, chemical, physical, and biological agents have been proposed according to the result to be obtained (reviewed in [100]).

Tissues from several animals source have been used for dECM decellularization including cow tendon, pig liver and heart, goat lung and kidney, and rat liver and lung. The pig represents the most used animal source since it showed physiological and anatomical feature close to human and also a short gestation period. However, in order to overcome infectious processes, the use of human-derived ECM appeared as suitable dECM for implantation. Human tissues, obtained from cadavers or from donated tissues of human tissue Biobank, were successfully used for preparing dECM. Due to the limitation of biocompatible autologous grafts and batch variability, recently plant tissues were indicated as a valid alternatives for scaffold preparation [101]. The surface area, the system of interconnected pores, the various degree of hydrophilicity, and biomechanical properties render it appropriate for the characteristics of plant tissue [101]. It is worth noting that, human cells in culture aligned along with the plant microstructure which underlies the relevance of topographical features of the scaffolds [102].

Finally, the next generation of dECM is represented by bio-inks and bioprinting technology. In the case of dEMC, however, the bio-ink is the building blocks of the bioprinted dECM with the pivotal role to offer the suitable microenvironment to the encapsulated cells. In this case, the hydrogel of the bio-ink have the same components and act the same function of the ECM of the native and varies from tissue to tissue [103]. However, conventional hydrogels for bioprinting are not able to mimic the complexity of native ECM needed for cells engraftment.

## 5. Mechanical Properties of Biomaterials

There are numerous biomaterials for determining cell fate or helping the reprogramming process that can be classified for biomechanical properties.

**Figure 2:**
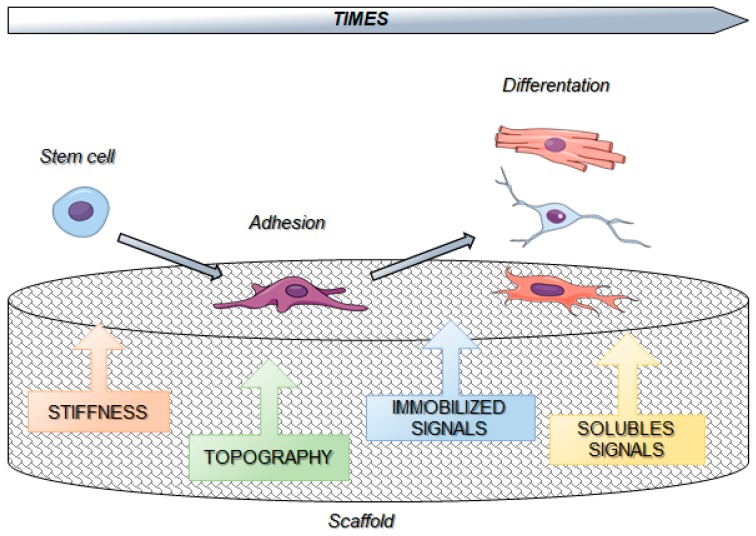
Biomaterial features affecting cell fate.

The development of engineering-based approaches allowed to obtain tuned modulation of extracellular signals by biomaterials and their use in cellular decision making (Figure 2). 

### 5.1. Viscoelastic Properties

Cells exhibit viscoelastic properties and as such, they yield typical characteristics of those materials, such as stress relaxation, creep, strain-rate sensitivity and hysteresis [104].

In particular, stress relaxation is the observed time-dependent reduction in stress when a material is subject to some strain. Creep is the opposite property, occurring when constant stress tends to elongate the material. Another property is the strain-rate sensitivity, which in general changes from tissue to tissue. Finally, the process of hysteresis occurs when the stress loading and unloading curves, depending on the force, differ substantially; the area between the two different curves thus indicates the loss of energy due to internal friction. In the recent years, many efforts have been made towards the understanding of the mechanobiology to control cell fate, using the latest technological platforms able to measure the biomechanical response of living cells to the microenvironment and their interactions. Among them, we can list atomic force microscopy (AFM) techniques and optical, magnetic, and acoustic tweezers [105].

Biomaterials designed for tissue engineering need to reproduce the same mechanical features of the native tissues determined by their viscoelastic behavior [106]. Moreover, the biomechanics’ substrate has to induce cell differentiation and to supply signals for ECM protein synthesis, allowing maintenance of the synthesized ECM proteins within the neo-constructs. It is indeed the structure of the different components of the ECM which determine the viscoelastic properties. The collagen fiber architecture of an ECM scaffold plays a critical role in determining its biomechanical behavior. The alignment and organization of collagen fibers are dependent on the function of the source tissue from which the ECM is derived. For example, the collagen fibers from a tendon are aligned along the long axis of the tissue and provide excellent resistance to strain. Therefore, the use of ECM from tendons is a good option for repairing ligaments [107,108,109]. The degree of alignment of the collagen fibers within an ECM scaffold changes as the scaffold is loaded. 

It is possible to predict the physical and mechanical properties of an ECM on the basis of its collagen fiber architecture. The collagen fiber alignment of a scaffold and the methods to modify the collagen fiber alignment may also be used to design scaffolds with desired mechanical properties [110]. 

The methodologies used in mechanobiology research [105] are different and include the well-established AFM technique, used to provide a topographic picture of cells and biomolecules and to describe the dynamical properties of the biomaterial. For instance, this is used to determine the molecular networking need for cell adhesion to a biomaterial. Tweezing techniques allow measuring both cellular stiffness and intracellular mechanisms. Thanks to the major advances in microfluidics, dynamical biomechanics aspects, and mechanotransduction mechanisms can be investigated in a 3D-like framework. Finally, traction force microscopy (TFM), with the help of a confocal microscope, has been exploited in order to measure stress on the cell surface, as well as for identifying 3D traction and stress areas of single cells within elastic and viscoelastic biomaterials. Such technology can lead to future development in the area to study synthetic and natural fibrous gels (such as collagen), in such a way that they mimic the physiological, biophysical and biochemical parameters of the microenvironment. Nevertheless, few methods are currently available for powerful and exact modeling that can describe cell behavior, rising the problem that many cell assays are needed to identify best biomechanical features of the biomaterial. A computational framework for creating a multi-component cell model was recently developed, called the “virtual cell model” that displays the feature for predicting changes in whole cell and cell nucleus characteristics (in terms of shape, direction, and chromatin conformation) on different substrates with different biomechanical features [111]. The Authors demonstrated that using this model was possible to predict cell and nucleus geometry on grooved substrates; in fact, virtual cell elongated along the grooves leading to modifications in nuclear morphology probably due to the propagation of stress from the external membrane to the nucleus by the viscoelastic cytoskeleton and cytoplasm. Another study has reported that changes in cellular shape determined strong effects on the nuclear shape and structure, resulting in chromatin condensation [112] leading to an alteration of the spatial configuration of chromatin, thus determining modifications in cellular fate and behavior. The virtual cell model was also used to predict the cell response to ECM elasticity [111] by employing 3D-elastic networks with triangular shapes. The Authors showed that a stiffer ECM was responsible for an increase in nucleus spreading. Also, localized shape deformation of the nucleus was associated with a higher substrate stiffness.

### 5.2. Effects of Stiffness 

The elastic moduli in the body tissues vary from <1 kPa in the brain to >1 GPa for bone [113,114,115], whereas in the majority of noncalcified tissues is generally below 50 kPa. The first report indicating that mechanical cues alone modulate cellular spreading and motility were reported by Pelham and Wang (1997) [116] in different cell types with effects dependent on cell type [117,118]. The effect of stiffness has been examined mainly in natural materials such as collagen, but the study of stiffness without considering other properties of the materials such as ligand density is difficult in natural materials. Therefore, many studies pointed in the use of synthetic materials such as polyacrylamide (PAA), PolyDiMethylSiloxane (PDMS), PolyethyleneGlycol (PEG), or polysaccharide-based biomaterials such as alginate or hyaluronic acid (HA), which become cell adhesive by introducing ECM components or adhesive peptide sequences. PAA gels modified with collagen were reported to induce MSC differentiation dependent on the elasticity of the substrate [2]. It is surprising that the authors reported differentiation patterns matching those of native tissue in depending on stiffness, i.e., with a low level of stiffness they obtained the neural phenotype expression, while at higher levels they obtained the osteogenic one. Other authors reported that these differences are not attributable only to stiffness levels but also to variations in collagen conjugation [119]. In fact, by modulating PAA gel formulations to obtain different stiffnesses, a change in the pore size was achieved, altering the spacing between collagen conjugation points and potentially affecting the mechanic information induced by the collagen to the cells. The relation between collagen linkage site spacing, stiffness, and stem cell differentiation remains unclear, although subsequent studies have used different approaches to study these variables and have obtained different results [120]. However, these findings indicate that separating the role of stiffness from other variables is complex, even in two-dimensional (2D) cultures. However, many cells in vivo grow in three dimensions (3D).

### 5.3. 2D and 3D Hydrogels

To study the role of stiffness in such environments, materials that are compatible with cell encapsulation were used. Hydrogels are the most generally used materials that merge water absorbency, retention, and controllable release, which allows them to be engineered in any shape and size. At the macroscopic level, they are extensively employed as biosensors and for drug delivery, while at the microscopic level, there is extensive use of hydrogels in order to study cell-ECM interactions and mechano-transduction [121].

Recently, the effects of stiffness gradient substrates like physical cross-linking and photopolymerization on cell behaviors have been thoroughly studied. Assessing the hydrogel’s mechanical properties reveals some difficulties due to their ability to deform and absorb/retain water. However, hydrogel’s mechanical properties were evaluated by using tensile, compression and bending tests and more recently also through ultrasound and puncture tests [121]. Hydrogels show good biocompatibility due to their humid, soft surface; thanks to that they decrease stimulation and friction with nearby tissues. The hydrogel stiffness modulates cell behavior and may be proposed as a multidimensional platform for culturing able to mimic cell microenvironment. Using HA modified with Arginylglycylaspartic acid(RGD) peptides it was showed that MSCs display differences in cell shape and spreading depending on both the stiffness of the material and the dimensionality of the matrix [122].

Advances in biomaterials allow designing 3D hydrogel culture systems. Hydrogel-based 3D culture model is able to mimic tumor-like features, giving more information on cancer cell behaviors which cannot be observed in usual 2D cultures. Hydrogels have also been used to mimic ECM mechanical properties, especially stiffness in both 2D and 3D frameworks. However, besides the ECM stiffness, its gradient also has a pivotal role in both physiological and pathological aspects. The advantage of using gradient stiffness hydrogels lies in the fact that they can simulate natural ECM stiffness gradients on a single biomaterial. Their design is at the basis for extensive research on cell-ECM interactions, cellular impairment and disease [121]. Cells which were observed to disperse more on stiffer 2D substrates showed biphasic response with spreading over 3D gels. The techniques used to design gradient hydrogels include physical cross-linking and photopolymerization, as well as 3D printing and lithography. On the other hand, the mechanical properties of the designed gradient hydrogels must be validated in order to meet different research requirements. Standard assessing methods include nanoindentation, tensile, compression, magnetic drive, ultrasound and puncture testing [121]. Gradient stiffness hydrogels were largely exploited for ECM simulation and biosensor design. They have also been used to study cell mechanical properties and drug delivery.

This kind of hydrogels gives new options for the study of efficiency in biomechanics and help the design of hydrogel-based biomaterials for tissue engineering, therapeutic and diagnostic tools. One of the limits of hydrogels is that the adhesion is mediated by short peptides that are not similar to ECM in transmitting the forces. Chen and colleagues by using methacrylated dextran functionalized with RGD electrospunned into fibers of different diameters, density, and anisotropy, and cross-linking, and modulating the stiffness, analyzed how stiffness changes affect cell fate [123]. At lower stiffness level, a local increase of RGD density was observed, leading to increased proliferation and migration. Notably, stiffness is not constant over time in tissue and may suffer important changes during pathological conditions such as cancer [124]. Several authors have set up different materials to analyze how cells respond to dynamic changes in stiffness. These materials enable cells to grow at one stiffness level for some time before that the stiffness level of the material starts changing. For example, by using methacrylated HA with different cross-linking reactions [125], MSCs grow on these gels, displayed augmented area and a differentiation fate towards osteogenic lineage with dynamic stiffening. The use of materials that can be softened or stiffened without changing polymer chemistry is useful to study the importance of dynamic changes. For this purpose, alginate gels with CaCl2 or a chelator included in liposome containing gold nanoparticles were produced. In this way it was possible to control the stiffness of the material. When fibroblasts were cultured in gels that stiffened, they were observed to change their morphology from an amoeboid shape to rounded.

In order to study the impact of mechanics, materials developed with embedded liposomes containing gold nanoparticles were utilized [126]. When exposed to light, the heat from the nanoparticles released the liposome cargo and this caused an increase or decrease in material stiffness. One limitation of this system is that a single liposome formulation is used to both soften and stiffen the gel, preventing reversible patterns from being tested. As an alternative, PEG cross-linked with azobenzene containing cross-linking peptides can be softened or stiffened by the use of light to convert azobenzene between cis and trans configurations [126]. Development of these systems may lead to an improved understanding of how changes in tissue mechanics influence cellular decisions during the dynamic processes of development and disease progression.

The stiffness of a substrate for culturing strongly influences cell morphology and stability [116]. Generally, cells grown on rigid materials have numerous stable focal adhesions connected to the substrate. On the other hand, cells grown on soft materials displayed disorganized cytoskeleton and dynamic focal adhesions [127].

### 5.4. 3D Fibrillar Matrices

3D fibrillar matrices are broadly utilized in tissue engineering and for biophysical studies, in particular, fibrillary assemblies of collagen. The structural and mechanical characteristics of these structures depend on factors, including temperature, protein concentration, ionic strength, and pH [128]. Chemical variations of collagen fibrillar protein with cross-linking agents ameliorate mechanical properties, including stiffness and porosity, and biocompatibility. Nevertheless, cross-linking decreases the natural properties of collagen, such as low immune response, low toxicity, and the capability to promote attachment and cellular growth [129]. 

Fibrillary assemblies of collagen create different tissue scaffolds, including tendons, blood vessels, basal membranes, cornea, ligaments, and skin [74]. Interestingly, the 1,4-dioxane solvent may represent a potential use on collagen enrich matrices, such as pericardium, and could be a co-adjuvant or a substitute to the conventional glutaraldehyde cross-linking techniques [128]. In particular, pericardium treated with 1,4-dioxane showed thermal, structural, and biological properties significantly enhanced compared to the native pericardium [128].

Other in vitro “polymerizable” alternatives include fibrin, and Matrigel, and in vivo-like 3D cell-derived matrices, with different physical properties. 3D cell-derived matrices are closer to in vivo conditions during embryonic development [130]; they generally comprehend fibronectin, but also hyaluronic acid, collagens, and other proteins, and consist of highly organized, robust fibrillar matrices that promote 3Dmatrix adhesion and directional cell migration [131]. Contrary to collagen and fibrin that are usually single protein polymerized in a 3D random matrix, Matrigel consists of a gel without distinct fibers [131]. Really, distributed force along a direction induces fiber alignment along the axis of force, whereas the other fibers experience different degrees of buckling or compression, which decreases the “bulk” mechanical characteristic of the gel [132]. These mechanical properties allow a single cell to locally be part of a matrix as soft or stiff, basing on the tension generated to a specific fiber (if parallel is rigid if perpendicular is soft). The alignment of cell adhesion with a fibrillar matrix is related to the adhesion size in collagen gels or on electro-spun fibers, indicating a mechanical response to the fiber stiffness along its length [133]. Soft or stiff substrates depends on mechanotransduction processes at nuclear and adhesion levels, influencing cell differentiation patterns [134].

The molecular mechanisms at the base of cell response to different stiffness is still unclear, even if some studies indicated that it is related to differential expression of anchorage proteins. However, other reports indicate that the response of cells to stiffness is independent of anchorage proteins [119]. Interestingly, YAP/TAZ nuclear/cytoplasmic ratio increased with stiffness in 2D but decreased with stiffness in 3D gels, indicating that studies on the mechano-transduction signaling may be useful to understand cellular decisions in these different conditions [122].

However, what is clear is that soft or stiff substrates differently result in mechanotransduction processes at nuclear and adhesion levels, influencing cell differentiation patterns [2,135].

### 5.5. Effects of Topography 

Regarding topography of the substrate, there are still few studies that unequivocally demonstrate which combination of parameters favors or prevent cell adhesion.

Many pieces of evidence indicate that a combination of dimensions influences focal adhesion dynamics, suggesting a possible common mechanic response of cells to topographic parameters of substrates. It has been shown how the geometry of the substrate may affect focal adhesion orientation as well as their formation [136]. Focal adhesions orientation determines cytoskeletal dynamics orienting stress fiber. This event is defined “contact guidance” [137] and depends on the topographic characteristics of the material since too wide or too close ridges do not determine efficient confinement of focal adhesion, thus counteracting the guidance activity [136,138]. On this basis, material features may be adapted to affect focal adhesion dynamic and formation by inducing signaling proteins or modifying cytoskeleton structure and contractility [136]. The pivotal role of ligand density and patterning topography and roughness or material stiffness is believed to influence cell behavior ad is considered to mimic ECM features. 

Several studies have performed a systematic analysis of the responses to topographic cues on a large number of cell types. Among these, as reviewed by Ventre et al., replica folding, nanoimprint lithography, block copolymer micelle nanolithography show a good match between features fidelity, resolution, and large area patterning [139]. Studies on dynamics require the design of materials responsive to the stimuli, together with actions that make the material efficient and biocompatible. Application of dynamic topographies, i.e., changing shape and height, requires the coordinated motion or matter removal on a submicro- or micro-scale level [136]. 

## 6. Biomaterials and Cell Reprogramming

Natural and synthetic biomaterials have the ability to connect and coordinate with the biological system, and when they are executing their functions, they require not to be damaging and unsafe for the patient. In the field of regenerative cell therapy, it is extremely important to regulate cell fate involving stem cell and reprogramming of mature cells. Reprogramming process needs to be accurately monitored and regulated, otherwise if the process in not sufficiently supervised, the cure might lead to teratoma, and not to the treatment for the patient. In this context there are many biomaterials that can support and cooperate in the reprogramming process, in fact biomaterials are able to supply microenvironments that simulate the exact cell compartment, favoring cell differentiation toward specific cell types [140]. An intensive examination of the bidirectional interplay between cells and compartments is fundamental to engineer next-generation biomaterials, in order to properly direct cell reprogramming in expected behavior. There are three main alternatives for the transition of a cell type into another: the direct reprogramming that consists in the conversion of a somatic cell into a different one; the direct reprogramming into a stable pluripotent stem cell line succeeded by the directed differentiation; indirect reprogramming obtained with a transient induction of a partly reprogrammed cell, and then the differentiation into the expected somatic cell type. The ability of direct cell reprogramming gained a great interest in the fields of basic research and regenerative medicine. For the first time, in 2006, Takahasaki and Yamanaka demonstrated that the combo of four transcription factors Oct4, Sox2, Klf4, and c-Myc are able to reprogram any cell to the pluripotent state and they called them induced pluripotent stem cells (iPSC) [141]. Since this discovery, direct reprogramming has come out as a new and important process to manage cell fate, in fact overexpression of several factors, i.e., general reprogramming factors or lineage-specific transcription factors, are responsible for the cell fate change of already differentiated cells. Contextually, biomaterials are able to supply biochemical signals or physical and topographical stimuli on cells, that can guide and influence the cell fate reprogramming [140]. More in-depth, biochemical signals such as ECM components, growth factors, and other molecules are essential for the control of cell fate and reprogramming, through the activation of integrins and other downstream signaling mechanisms [142]. Collagen, laminin, and fibronectin are among the most important ECM components. 3D collagen gel has been used to obtain CNS stem cell differentiation towards functional neural cells, which have the characteristic to not attach to synthetic hydrogels, so the latter need to be modified with ECM molecules as collagen. Instead, stem cells captured into biologically collagen hydrogel are able to spread in their medium with the addition of bFGF, developing neurons, astrocytes and oligodendrocytes. In a study about the impact of the matrix structure of 3D scaffolds on embryonic stem cell differentiation, it has been demonstrated that embryonic body development is dependent on the different amount of collagen [143]. With high collagen concentrations, they observed inhibition of cellular apoptosis, with consequent disruption of embryonic body formation. The presence of fibronectin in 3D collagen scaffolds allowed the stimulation of endothelial cell differentiation and vascularization. In contrast, laminin increased the capacity of embryonic stem cells to differentiate into functional cardiomyocytes. In another work it has been observed that endothelial progenitor cells were able to reprogram to smooth muscle cells, with a consequent assembly of blood vessel structure using a 3D dense collagen gel [144]. Fibrin is formed by the combination of thrombin and fibrinogen. It has been demonstrated that the concentration of fibrinogen is more important than thrombin concentration in the design of an ideal scaffold [145]. In contrast a higher concentration of thrombin is responsible of inhibition of cell migration in the fibrin scaffold. In addition, increased cell density induces stronger cell growth and differentiation. In order to hamper the fibrin degradation, the scaffold has been modified with PEG, which enhanced the performance of embryonic stem cells differentiation and reprogramming [146]. Furthermore, Ingram DA and colleagues obtained vessel-like structures employing endothelial progenitor cells isolated from human umbilical cord blood [147]. They used PEG associated with bioactive peptides, and the combination of EPCs and PEG hydrogels conferred the ability to create 3D microvessel structures without the addition of other angiogenic growth factors. Growth factors are very important among the biochemical signals for cell reprogramming. Indeed, growth factors are not able to change cell fate in a direct process, but for sure they can influence it. For instance, it has been published that Activin-A or TGFβ1 influences the differentiation of mesodermal cells; bFGF, BMP4, EGF, or retinoic acid encourages ectodermal and mesodermal differentiation, while NGF and HGF induce all three embryonic germ layer differentiations [148]. Moreover the notch signaling pathway is involved in the regulation of epithelial differentiation, in fact the presence of the notch ligand Jagged-1 (in a bound form) on the biomaterial surface, together with the binding to Polystyrene or poly(hydroxyethyl methacrylate, HEMA), helps differentiation of stem to epithelial cells [149]. In a recent work it has been showed that notch signaling biomaterials act through a time-specific activation process, leading to the promotion of ectodermal gene expression, and the presence of Jagged-1 influenced the differentiation of cardiovascular progenitor cells to cardiomyocytes [150]. In addition, recent studies have identified YAP and TAZ transcription factors as two of the major regulators of mechanotransduction correlating the biomechanical microenvironment and cell fate [97]. In fact, YAP/TAZ are fundamental for the differentiation of MSCs influenced by ECM stiffness and for survival of endothelial cells controlled by cell shape [151]. Moreover, it has been published that YAP nuclear localization coadiuvates with stiff surfaces in facilitating cell proliferation [30]. Conversely, flexible matrices prevent from nuclear localization of YAP, and cannot encourage cell proliferation, and consequently they induce differentiation. The interaction between YAP and TEAD transcription factors promotes cell cycle progression, and YAP depletion is responsible of the neuronal differentiation [152]. These findings suggest that YAP/TAZ are important mediators of mechanotransduction and its interplay between microenvironment and cell behavior. Furthermore, by using a combination of small chemical components, which do not present immunogenicity, are able to permeabilize cells and are cost-effectiveness, it is possible to influence cell fate and reprogramming [153]. In this regard, studies from recent years discuss the use of CHIR99021, SB431542, ISX9, and Forskolin to promote functional neurons reprogramming from mouse fibroblasts [154], and the use of nine different chemical molecules to induce cardiomyocytes differentiation from human fibroblasts [155]. Together with biochemical aspects, mechanical strength and surface topography are two of the main physical features of biomaterials in cellular reprogramming. Initially, cell fate is determined by the elasticity of the matrix in the microenvironment, and in this regard, usually stiffer substrates lead to stiffer cells, and softer substrates lead to softer cells [156]. Through the modulation of matrix elasticity, Engler and colleagues demonstrated that human MSCs go through neurogenic differentiation from elastic modulus in 0.1–1 kPa, myogenic differentiation in 8–17 kPa, in osteogenic differentiation in 25–40 kPa [113,157]. In the interplay cell-substrate also stiffness can strongly regulate cell fate, in fact the use of soft hydrogel biomaterials contributes to the generation of iPSCs, promoting the biological process of mesenchymal-epithelial transition [158]. Another important physical characteristic that can influence cell reprogramming is the fiber diameter of substrates [159,160]. It has been observed that laminin covered with polyethersulfone (obtained by electrospinning) is able to regulate neural stem cell differentiation, in fact decreasing the fiber diameter, there is an increase of neural stem cell proliferation rate. Even cell morphology is influenced by the fiber diameter, in fact depending on the size limitations cells exhibit different arrangements: in small fiber they appear in a stretched and multi-directional shape, while in large fibers they organize themselves in an extended form along a single axis. Moreover, it has been demonstrated that among the physical properties of biomaterials in cell reprogramming, the pores of scaffold can influence the cell adaptation, dissemination and tissue organization [161]. Levenger et al. composed 3D porous Poly(lactic-co-glycolic acid) (PLGA) and poly(L-lactic acid) (PLLA) scaffolds with 250-500 μm pores in order to help the embryonic stem cell differentiation toward the expected cell, with the addition of appropriate growth factors i.e., TGF-β for cartilage, Activin-A for liver-like cells, and RA for neuroectodermal like structures. In addition, there are many evidences on the influence of topographical features on direct cell reprogramming process. In this regard Leong and colleagues demonstrated that the interaction between cells and topography can promote direct reprogramming from fibroblast to induced neurons, confirmed by overexpression of the neuronal transcription factors Asc11, Brn2 and Myt1L [162]. The rearrangement of the cytoskeleton, influenced by substrate topography and stiffness, through a FAK- and myosin II-dependent mechanism, is a pivotal step during the neural induction [163]. Considering all the physical aspects also biomaterial-based microparticles have been considered for encapsulations and releasing several molecules in many biomaterial experiments [164,165]. Gelatin, agarose and PLGA, used as materials for microparticles, showed stability and integration with embryonic stem cells used for the experiments [166]. However, this procedure revealed a remarkable pitfall, due to the low efficiency of the mixing process between microparticles and stem cells, requiring forced aggregation to obtain the maximal production of expected differentiated cells. Based on these evidences, indeed physical aspects of cell environments are fundamental in determining cell fate and reprogramming. Thus, based on these considerations, both biochemical and physical stimuli, responsible of matrix stiffness regulation and protein concentration on the substrate, is a crucial and imperative approach to influence cell fate and reprogramming. 

## 7. Biomaterials and Stem Cells Interactions

Thanks to their self-renewal and pluripotency ability, stem cells are a unique class of cells that can differentiate toward almost all cell types of a particular organ or tissue. In recent years, stem cell research has gained remarkable progress in many fields, including regenerative medicine and tissue engineering. Stem cell-based therapies can contribute with innovative solutions for the treatment of many diseases, but still, there are a few obstacles to overcome, due to the unhampered differentiation and functional rejection of implanted tissues. In this regard, the handling of stem cell fate is one of the most relevant aspects to control in regenerative tissue engineering and medicine. Commonly, stem cells can be divided into embryonic stem cells (ESCs) and adult stem cells. ESCs are able to promote the differentiation into any cell types of the ectoderm, the mesoderm, and the endoderm. Due to the many ethical issues about the origin of ESCs, nowadays in the field of regenerative medicine, there is a strong propensity, toward the use of induced pluripotent stem cells (iPSCs) thanks to their genetic characteristics that resemble ESC-like state or MSCs [167]. However, in relation to implantation in vivo, ESCs and iPSCs need to be directed toward a specific lineage-differentiation and implantation or injection to escape from the possible development of a teratoma tumor, that generally occurs when ESCs and iPSCs remain in an undifferentiated state for long [168]. Moreover, considering the potential of pluripotent differentiation typical of ESCs and iPSCs, it would be problematic to direct differentiate them into specific lineage of cells [167]. For all these reasons, researchers tend to prefer the use of adult stem cells because they show minimal tumorigenicity and they do not present ethical dilemmas to consider. In recent years, there is a rising interest in the study of mechanisms of interactions between stem cells and biomaterials. Stem cells, during their growth, are subjected to several physical signals, that include elastic, compressive, osmotic or fluid stresses, occurring into biochemical interactions with the ECM. There are many pieces of evidence on the fact that mechanical stimuli can strongly affect stem cell growth and differentiation. In fact, a tensile stress of the ECM can provoke stretch of the cytoskeleton and nucleus through focal adhesion, whereas compressive stress of the ECM can greatly modify ion concentration and local charge density, activating sensitive ion channels [156]. Moreover, the identification of several types of integrins and cell surface proteins have clarified the molecular mechanisms activated during cell-biomaterial interactions, as described above. For example, in a recent work Kanninen and colleagues found that the two ECM components, laminin-511 and laminin-52, could be utilized as matrices for the creation of a highly defined scaffold to activate stem cell differentiation into hepatic tissue [169]. There are several limits for the detection of quantitative and detailed information on the interactions between stem cells and biomaterials because they occur in the nanoscale and in physiological conditions. In order to overcome these limitations and to find a better method for a quantitative detection, experiments of single-cell force spectroscopy (SCFS) have been performed, using micropipettes [170], magnetic-[171] or optical tweezers [172]. However, these methods are characterized by a low force resolution (from 10pN to 1nN). Alternatively, the atomic force microscope (AFM)-based force spectroscopy grants a better spectrum of measurable forces (between 10 pN and 100 nN). This method is able to analyze the communication that elapses between cancer human cell and biomaterials [173,174], attaching a cell to the AFM cantilever and then probing against the materials attached to the substrate, but is not applicable to fragile cell lines that cannot survive as single cells. The interplay between biomaterials and stem cells is responsible of the influence of stem cell properties both in vivo and in vitro, in dictating stem cell fate [175]. Following these principles, thanks to the recent advances in stem cell research in tissue engineering and regenerative medicine, the key step is to produce a more physiologically relevant scaffold system in vitro, looking at mechanical strength, durability and biocompatibility, that can mimic as good as possible the stem cell microenvironment, in terms of viability, cell attachment, self-renewal, migration and differentiation. In this respect, it is fundamental to manipulate the properties of materials surrounding stem cells, such as stiffness, surface topography, density or porosity [176,177].

## 8. Conclusions and Future Directions

Adhesion to substrate generate signals regulating several features of cell behavior, including migration, proliferation, and differentiation. Therefore, focal adhesion-cytoskeleton dynamics-contractility represents a unit where a change in one element determines a modification in the other affecting cell behaviors and fates. 

Recent experiments have shown that the biomechanical features of a cell determine how the microenvironmental information (such as tissue compression, growth factors, or cytokines) are processed for the gene expression control Several findings have revealed the crucial role exerted by chromatin conformation in the integration of transcription factors and chromatin remodeling enzymes in the regulation of gene expression [53,178]. 

In this regard, biomaterials may have a crucial role in affecting cell reprogramming and fate by triggering gene expression. 

So far, static platforms relevantly contributed to getting more insight into understanding the best combinations of signals to obtain a specific response. On the other hand, static platforms are limited in answering all the aspects of cell dynamics [136]. Resolving all the open questions on this issue may be useful for the developing of new and efficient culturing systems but they also can clarify how the microenvironment may affect cell fate and cell reprogramming. 

Several reports have indicated that cell behavior and fate are not disseminated only by a single signal, but also by a complex network of several signals operating on different length- and time-scales that determine cell fate [136,179,180]. For this reason, specific materials endowed by specific switches should be designed for acting signals at different timepoints. However, changes in the ECM are not restricted to adhesion signals and implicated different signals in agreement with different chronologies. Indeed, the published research and reports regarding a dynamic platform mainly concern platforms regulating adhesion/detachment and migration. Furthermore, high potential of the cell dynamics is reported, mainly in relation to cell fate and in the acquisition of specific tissue phenotypes [181,182,183]. In the design of more efficient and innovative culturing systems, the parameter “time” is of considerable importance, but the dynamic surfaces cannot acquire the complex characteristics of the native ECM. An interesting approach to overcome this issue has been recently projected by Hay et al. who brought the idea of bacteria-based materials [184]. These finely changed the adhesive microenvironment upon external directions, ultimately regulating stem cell fate decision.

Therefore, the field of the dynamic platform is expected to solving complex biological issues, including migration and differentiation, but it could also impact the bio-pharmaceutical areas due to the design of devices for drug screening and tissue regeneration. Finally, it has to consider that while 2D surfaces are useful to follow the behavior of the epithelial and endothelial cell, most of the biological events occur in a 3D ambience. Laser two-photon or holographic techniques might represent useful instruments for reaching a spatial control of the chemical/physical properties of the 3D environment with a good resolution [136,185,186]. Nevertheless, integrating diverse stimuli in 3D reaching control on single adhesion points still need additional studies. 

In summary, cell reprogramming is a potential medical procedure for drug development, disease modeling, degenerative diseases, and other pathologies. Biomaterials are able to render more stable and effective in this procedure. However, there are some issues to overcome. Regarding cell reprogramming, the safety needs to be verified in long-term clinical trials, and the efficiency needs to be improved to increase the number of cells necessary for clinical approaches. As concerns the biomaterials, designing a fully synthetic reprogramming microenvironment allows avoiding the immunoreactivity and improving the quality. Combining natural and synthetic biomaterials represents an important strategy in bioengineering, but there is a lack of biomaterials and biostructures with clinical applicability. For clinical application, the problems regarding low efficiency, treatment convenience and time consuming should be considered together. Moreover, synergistic efforts from the field of biomaterial science, bioengineering, medicine, and cell biology are necessary to overcome these issues and, with the help of cell reprogramming, rapidly increase the biomaterial potential in tissue regeneration. Overall, designing innovative biomaterials and understanding the biomaterial biomechanical properties will be useful in improving the regenerative medicine, creating human tissue alternatives, able to mimic the native tissue structure and function.
